# How do species, population and active ingredient influence insecticide susceptibility in *Culicoides* biting midges (Diptera: Ceratopogonidae) of veterinary importance?

**DOI:** 10.1186/s13071-015-1042-8

**Published:** 2015-08-28

**Authors:** Roger Venail, Jonathan Lhoir, Moussa Fall, Ricardo del Río, Sandra Talavera, Karien Labuschagne, Miguel Miranda, Nonito Pagès, Gert Venter, Ignace Rakotoarivony, Xavier Allène, Bethsabée Scheid, Laëtitia Gardès, Geoffrey Gimonneau, Renaud Lancelot, Claire Garros, Catherine Cêtre-Sossah, Thomas Balenghien, Simon Carpenter, Thierry Baldet

**Affiliations:** EID Méditerranée, 34184 Montpellier, France; Cirad, UMR15 CMAEE; INRA, UMR1309 CMAEE, 34398 Montpellier, France; Institut sénégalais de recherches agricoles (ISRA), BP 1386 Dakar, Senegal; Laboratory of Zoology, University of the Balearic Islands (UIB), Km 7.5, Palma de Mallorca, Spain; Centre de Recerca en Sanitat Animal (CReSA), UAB-IRTA, Campus de la Universitat Autònoma de Barcelona, 08193 Bellaterra, Barcelona Spain; Agricultural Research Council – ARC- Onderstepoort Veterinary Institute, Private Bag X5, Onderstepoort, 0110 Republic of South Africa; The Pirbright Institute, Vector-borne Viral Disease Programme, Woking, GU24 0NF UK

**Keywords:** *Culicoides imicola*, *Culicoides obsoletus*, *Culicoides nubeculosus*, Insecticide susceptibility, Pyrethroids, Organophosphates, Vector control

## Abstract

**Background:**

*Culicoides* biting midges are biological vectors of internationally important arboviruses of livestock and equines. Insecticides are often employed against *Culicoides* as a part of vector control measures, but systematic assessments of their efficacy have rarely been attempted. The objective of the present study is to determine baseline susceptibility of multiple *Culicoides* vector species and populations in Europe and Africa to the most commonly used insecticide active ingredients. Six active ingredients are tested: three that are based on synthetic pyrethroids (alpha-cypermethrin, deltamethrin and permethrin) and three on organophosphates (phoxim, diazinon and chlorpyrifos-methyl).

**Methods:**

Susceptibility tests were conducted on 29,064 field-collected individuals of *Culicoides obsoletus* Meigen, *Culicoides imicola* Kieffer and a laboratory-reared *Culicoides nubeculosus* Meigen strain using a modified World Health Organization assay. Populations of *Culicoides* were tested from seven locations in four different countries (France, Spain, Senegal and South Africa) and at least four concentrations of laboratory grade active ingredients were assessed for each population.

**Results:**

The study revealed that insecticide susceptibility varied at both a species and population level, but that broad conclusions could be drawn regarding the efficacy of active ingredients. Synthetic pyrethroid insecticides were found to inflict greater mortality than organophosphate active ingredients and the colony strain of *C. nubeculosus* was significantly more susceptible than field populations. Among the synthetic pyrethroids, deltamethrin was found to be the most toxic active ingredient for all species and populations.

**Conclusions:**

The data presented represent the first parallel and systematic assessment of *Culicoides* insecticide susceptibility across several countries. As such, they are an important baseline reference to monitor the susceptibility status of *Culicoides* to current insecticides and also to assess the toxicity of new active ingredients with practical implications for vector control strategies.

**Electronic supplementary material:**

The online version of this article (doi:10.1186/s13071-015-1042-8) contains supplementary material, which is available to authorized users.

## Background

*Culicoides* Latreille (Diptera: Ceratopogonidae) are small haematophagous insects implicated worldwide as primary biological vectors of arboviruses causing important diseases of livestock [1]. These arboviruses include bluetongue (BTV), African horse sickness (AHSV), epizootic haemorrhagic disease (EHDV) and Schmallenberg (SBV) viruses [2]. *Culicoides-*borne arboviruses have a severe economic impact through direct loses due to the morbidity and mortality that occurs in susceptible animals. Additional losses also occur, however, due to the imposition of animal movement restrictions to limit BTV spread that inhibit animal trade [3, 4] and the indirect costs of monitoring and surveillance measures during outbreaks. *Culicoides* are also notorious as a biting nuisance in some regions, causing discomfort in humans and livestock and seasonal recurrent allergic dermatitis in horses [5–7].

In attempts to control *Culicoides*-borne arboviruses such as BTV and AHSV outside of their endemic range, compulsory vaccination campaigns and livestock movement restrictions are usually employed as the most effective way of controlling outbreaks [8]. Where safe and effective vaccines to *Culicoides*-borne viruses are either not initially available or economically unviable, control measures against *Culicoides* have been recommended by veterinary authorities to reduce host-vector contact and thus mitigate against arbovirus transmission. The use of insecticide residual spraying within stables and during transport when livestock is moved outside a restricted movement zone has been recommended in protecting animals with high economic value (e.g. prize rams and racehorses). Additional physical measures have also been suggested to reduce *Culicoides* populations such as the mechanical removal and/or reduction of larval breeding sites on farms and housing livestock during periods of high *Culicoides* activity [9].

To date, no insecticidal products have been authorized specifically against *Culicoides* in the European Union (EU), although a wide range of products are available, licensed and in use against other arthropods of veterinary importance [9]. Worldwide, the most commonly used method to protect livestock from *Culicoides* is the application of insecticides to livestock at risk of infection. Synthetic pyrethroid (SP) active ingredients are most often used in this role, but certain organophosphate (OP) products are also still available and licensed for use in Europe [7]. Insecticidal pour-on products exert their effect in two ways: primarily they are highly toxic to insects landing on the treated animal, often killing them within minutes of their landing on the host; secondarily they exert a contact irritation that may reduce the probability of the insect successfully initiating or completing a blood meal from the host. While some effort has been made to assess the efficiency of pour-on products against *Culicoides*, results vary greatly between studies according to different experimental designs [7]. Methodologies used include the exposure of *Culicoides* to hair clippings from treated animals [10–12] or direct exposure to a treated animal [13]. The variability of results across studies highlights the importance of using a standardized method to obtain comparable and reliable data.

Following the European Food Safety Authority’s recommendation to assess susceptibility of *Culicoides* to insecticides using standardized procedures [9], a World Health Organisation (WHO) standardized technique in adult mosquitoes has been adapted for use with *Culicoides* [13, 14]. This baseline information is essential for recommending the most effective insecticides and in detecting and monitoring the development of resistance. Insecticide resistance to earlier classes of insecticides including organochlorine-based larval treatments such as dieldrin and lindane was documented in *Culicoides* in the late 1950’s [15], but has not been examined for either OP or SP use. This risk exists considering that products based on single classes of insecticide have been used on a wide scale on livestock to control other arthropods including ticks, horn flies and stable flies in addition to often being used on crops.

Standardised information concerning the susceptibility of *Culicoides* to insecticides is at present limited to small scale studies [13, 14]. This study aims to assess the susceptibility of multiple populations of *Culicoides* species in different countries to the most frequently used insecticide active ingredients in Europe (SP: alpha-cypermethrin, deltamethrin and permethrin; OP: diazinon/dimpylate and phoxim) and Africa/Latin America (OP: chlorpyrifos-methyl). The main objective of the study is to generate reference baseline data regarding the efficiency of insecticidal products in killing *Culicoides* under laboratory conditions. Implementation of such insecticidal treatments into control programmes against *Culicoides*-borne diseases is then discussed.

## Methods

### *Culicoides* collection and identification

Susceptibility tests were performed on three *Culicoides* species. Laboratory-reared *Culicoides nubeculosus* Meigen were provided from a colony maintained in an insectary (temperature: 24 °C ±1 °C; relative humidity: 70 ± 10 %; light:dark: 12:12) at Cirad (Montpellier, France). This colony was established in Cirad during 2012 from eggs and larvae provided by The Pirbright Institute (UK). Field populations of *Culicoides obsoletus* Meigen and *Culicoides imicola* Kieffer were collected from multiple locations in two European countries (France, Spain) and two African countries (Senegal, South Africa) (Additional file 1: Table S1). Collection sites were privately owned farms characterized by abundant populations of *Culicoides* target species and reduced use of insecticides on the animals or pesticides on crops.

*Culicoides* were collected using a modified suction UV-light trap (OVI model, South Africa) [16] with the collection beaker replaced by a fine mesh netted cage to enable live collections. To prevent desiccation of *Culicoides* during the collection period, wet paper was placed on aluminium foil and rolled around the mesh cages. Traps were set before sunset and retrieved at dawn. *Culicoides* collection cages were stored in an isothermal container with an ice pack during transport to the insecticide trials room. Following completion of insecticide trials, field-collected individuals were morphologically identified to species level for *C. imicola* or to Obsoletus complex [17] using a binocular microscope and Obsoletus complex individuals were further identified to species level using a diagnostic multiplex polymerase chain reaction (PCR) assay [18].

### Selection of insecticides and production of impregnated papers

Insecticide active ingredients were selected from those used most frequently in pour-on formulations within Europe. All active ingredients were used at > 98 % purity (Pestanal®, a registered trademark Sigma-Aldrich Laborchemikalien Gmbh, London, UK). Test papers (Whatman n°1 filter paper, 90 g/m^2^, 12 × 15 cm) were impregnated following training provided by a WHO collaborative centre (LIN-IRD, France). Insecticide active ingredients were applied at different concentrations (Additional file 2: Table S2) to papers in a silicone oil as the carrier agent (2 ml per paper, 67 % acetone, and 33 % silicone oil). Control papers were impregnated with 2 ml of acetone-silicone oil mix only. Impregnations were conducted by the same person (RV) to ensure consistency. Papers were impregnated a few days before the testing period, wrapped in aluminium foil and then stored at 4 °C. Impregnated papers were sent to each country in a polystyrene cooler box with ice cooler packs for maintaining the temperature at 4 °C during transport. Each paper was used three times in assays and stored at 4 °C between trials.

### Insecticide susceptibility tests

Insecticide susceptibility tests were performed following the standardized WHO protocol for adult mosquito bioassay using test tubes (WHO/VBC/81.806) [19]) adapted for *Culicoides* [13]. Because insecticide susceptibility could be age specific [20], and physiological status and sex dependant [21, 22], bioassays were performed with 2–3 day old laboratory-reared *C. nubeculosus* unfed females. Due to the difficulties of colonizing *C. obsoletus* and *C. imicola*, adults were collected from the field and used one day after collection in bioassays. For field-collected *Culicoides*, only unpigmented females that were believed to have not previously taken a blood meal were used in data analysis, as determined through observation of abdominal pigmentation [23].

*Culicoides* were exposed for 1 h to either insecticide-impregnated papers or a control paper with the carrier compound only. For each replicate carried out on field-collected *Culicoides*, approximately 100 unsorted individuals were placed in each tube to obtain at least 25 unfed females of the target population. Following this exposure period, all *Culicoides* (including incapacitated individuals) were transferred using a motorised aspirator from exposure to observation tubes. Observation tubes were then stored vertically for 24 h and *Culicoides* within tubes were given access to a 10 % sugar solution provided on cotton wool pads through the top of the tube. Following the observation period, live and dead individuals were recorded and placed in 96 % ethanol. A replicate within the trials consisted of one complete set of serial dilutions and one negative control (untreated paper). Four replicates were performed for each active ingredient and target population. All susceptibility tests were performed in each country in a dedicated laboratory by the same trained person following standard protocols and at a temperature of 21 ± 3 °C and relative humidity of 70 ± 10 %.

### Statistical analysis

Dose–response analysis for *Culicoides* mortality followed WHO recommendations [22]. Mortality rates were calculated by pooling the total number of dead *Culicoides* by active ingredient concentration across all replicates and expressed as a percentage of the total number of exposed individuals. When control mortality exceeded 20 % of the *Culicoides* exposed the replicate was discarded, while at rates of 5 to 20 % control mortality, rates were corrected using Abbott’s method (corrected mortality = 100 x (% observed mortality - % control mortality)/(100 - % control mortality) [24]). Abbott’s method reduces the estimated mortality effect of the treatment by the non-treatment mortality, as measured by the control. Data were analysed by a probit regression analysis [25] using PriProbit ver. 1.63 to obtain susceptibility values (LC_50_ and LC_90_) and sigmoidal curves of dose–response estimations of each insecticide active ingredient for each target population.

A second insecticide susceptibility analysis was performed to determine the effect of species origin (country and population), active ingredient concentration and their interactions. The two families of insecticide active ingredients were analysed separately as the concentrations used in testing differed. Initially, a generalised linear model with a binomial distribution was fitted, leading to an over dispersion of data (goodness of fit, *p* < 0.05). To assess the fixed effects (species and origin), the differences in deviation between the complete model including fixed effects (species, origin and doses without interaction) and without the fixed effect were calculated, taking into account the dispersion factor. R freeware (R Development Core Team 2012) and additional packages (aods3, lattice) were used for data analysis and graphics [26].

## Results

A total of 29,064 unpigmented females were used in bioassays (11,761 *C. nubeculosus,* 11,975 *C. imicola* and 5,328 *C. obsoletus*). Among the 5,516 individuals collected belonging to the Obsoletus group: 5,328 (96.6 %) were molecularly identified as *C. obsoletus*, 152 (2.8 %) as *C. scoticus* and 36 specimens (0.6 %) were not identified and were excluded from the analysis. Amongst the 4,973 individuals from Obsoletus group collected in Corrèze, France; 4,815 (96.8 %) were identified as *C. obsoletus,* 149 (3.0 %) as *C. scoticus* and 9 (0.2 %) were unidentified; from the 543 individuals collected in Mallorca Island, Spain, 513 (94.5 %) were identified as *C. obsoletus*, 3 (0.5 %) as *C. scoticus* and 27 (5.0 %) were not identified. Data analysis was only performed with *C. obsoletus* as numbers of *C. scoticus* were not sufficient for examination*.*

Mortality recorded 24 h after 1 h exposure to insecticide active ingredients indicated that all *Culicoides* populations were susceptible to the active ingredients tested. The lethal concentrations (LC_50_ and LC_90_) calculated by probit analysis are presented in percentage of active ingredient (Tables 1, 2, 3, 4) and in its equivalent in mg/m^2^ (Additional file 3: Tables S3, Additional file 4: Table S4, Additional file 5: Table S5a, S5b). Within SPs, permethrin elicited the highest LC_50_ and LC_90_ values indicating less sensitivity, whereas deltamethrin gave the lowest values for all the populations studied. Among the OPs, chlorpyrifos-methyl produced the lowest LC_50_ and LC_90_ values, whereas diazinon gave the highest values.

**Table 1:** Susceptibility values (LC_50_ and LC_90_ expressed in % of active ingredient) of *Culicoides nubeculosus* from French colony to different active ingredients. Mortality was recorded 24 h after 1 h exposure to different concentrations

Active ingredient	No. test	LC_50_ %	LC_90_ %
	(*n*)	(95 % CI)	(95 % CI)
Deltamethrin	4	0.0003	0.0019
	(2,528)	(0.0001–0.0004)	(0.0012–0.0043)
Alpha-cypermethrin	3	0.0016	0.0199
	(1,883)	NA	NA
Permethrin	3	0.0102	0.1045
	(2,055)	(0.0080–0.0117)	(0.0849–0.1337)
Chlorpyrifos-methyl	4	0.0725	0.1661
	(1,803)	NA	NA
Phoxim	3	0.1532	0.2759
	(1,965)	(0.1143–0.2091)	(0.2036–0.5761)
Diazinon	4	0.1839	0.3317
	(1,527)	(0.0800–0.2747)	(0.2347–0.4333)

*No test* number of tests performed, *n* number of individuals tested, *CI* confidence interval, *NA* confidence interval not computed due to a large variability in the dose/response effect

**Table 2:** Susceptibility values (LC_50_ and LC_90_ expressed in % of active ingredient) of different populations of *Culicoides obsoletus* to different active ingredients. Mortality was recorded 24 h after 1 h exposure to different concentrations

Active ingredient	*C. obsoletus* (Corrèze, France)			*C. obsoletus* (Mallorca, Spain)		
	No. test	LC_50_ %	LC_90_ %	No. test	LC_50_ %	LC_90_ %
	(*n*)	(95 % CI)	(95 % CI)	(*n*)	(95 % CI)	(95 % CI)
Deltamethrin	3	0.0001	0.0008	4	0.0005	0.0032
	(1,491)	(0.0000–0.0002)	(0.0005–0.0018)	(382)	(0.0002–0.0011)	(0.0013–0.1246)
Alpha-cypermethrin	3	0.0012	0.0102			
	(502)	NA	NA			
Permethrin	2	0.0207	0.0668	4	0.0147	0.0840
	(527)	(0.0188–0.0229)	(0.0565–0.0822)	(131)	(0.0101–0.0203)	(0.0502–0.2445)
Chlorpyrifos-methyl	5	0.0182	0.0769			
	(615)	(0.0142–0.0222)	(0.0632–0.0980)			
Phoxim	6	0.0273	0.1177			
	(763)	(0.0238–0.0312)	(0.0944–0.1564)			
Diazinon	2	0.0848	0.2944			
	(917)	NA	NA			

*No test* number of tests performed, *n* number of individuals tested, *CI* confidence interval, *NA* confidence interval not computed due to a large variability in the dose/response effect

**Table 3:** Susceptibility values (LC_50_ and LC_90_ expressed in % of active ingredient) of different European populations of *Culicoides imicola* to different active ingredients. Mortality was recorded 24 h after 1 h exposure to different concentrations

Active ingredient	Corsica, France			Catalonia, Spain		
	No. test	LC_50_ %	LC_90_ %	No. test	LC_50_ %	LC_90_ %
	(*n*)	(95 % CI)	(95 % CI)	(*n*)	(95 % CI)	(95 % CI)
Deltamethrin	3	0.0002	0.0008	3	0.0003	0.0023
	(2,525)	(0.0001–0.0002)	(0.0005–0.0014)	(378)	(0.0002–0.0004)	(0.0016–0.0037)
Alpha-cypermethrin	4	0.0008	0.0034			
	(2.084)	NA	NA			
Permethrin	4	0.0194	0.0812	2	0.0175	0.1071
	(1.562)	(0.0171–0.0219)	(0.0695–0.0975)	(123)	NA	NA
Chlorpyrifos-methyl	1	0.0274	0.1273			
	(122)	(0.0176–0.0388)	(0.0784–0.3535)			
Phoxim	2	0.1053	0.2441			
	(2,207)	NA	NA			
Diazinon	2	0.1031	0.3424			
	(1,704)	NA	NA			

*No test* number of tests performed, *n* number of individuals tested, *CI* confidence interval, *NA* confidence interval not computed; due to a large variability in the dose/response effect

**Table 4:** Susceptibility values (LC_50_ and LC_90_ expressed in % of active ingredient) of different African populations of *Culicoides imicola* to different active ingredients. Mortality was recorded 24 h after 1 h exposure to different concentrations

	Rufisque, Senegal			Pretoria, South Africa		
Active ingredient	No. test	LC_50_ %	LC_90_ %	No. test	LC_50_ %	LC_90_ %
	(*n*)	(95 % CI)	(95 % CI)	(*n*)	(95 % CI)	(95 % CI)
Deltamethrin	4	0.0005	0.0015	3	0.0003	0.0020
	(458)	(0.0004–0.0005)	(0.0012–0.0018)	(291)	NA	NA
Permethrin	3	0.0031	0.0168			
	(521)	(0.0018–0.0045)	(0.0105–0.0431)			

*No test* number of tests performed, *n*  number of individuals tested, *CI* confidence interval, *NA* confidence interval not computed; due to a large variability in the dose/response effect

**Fig. 1:**
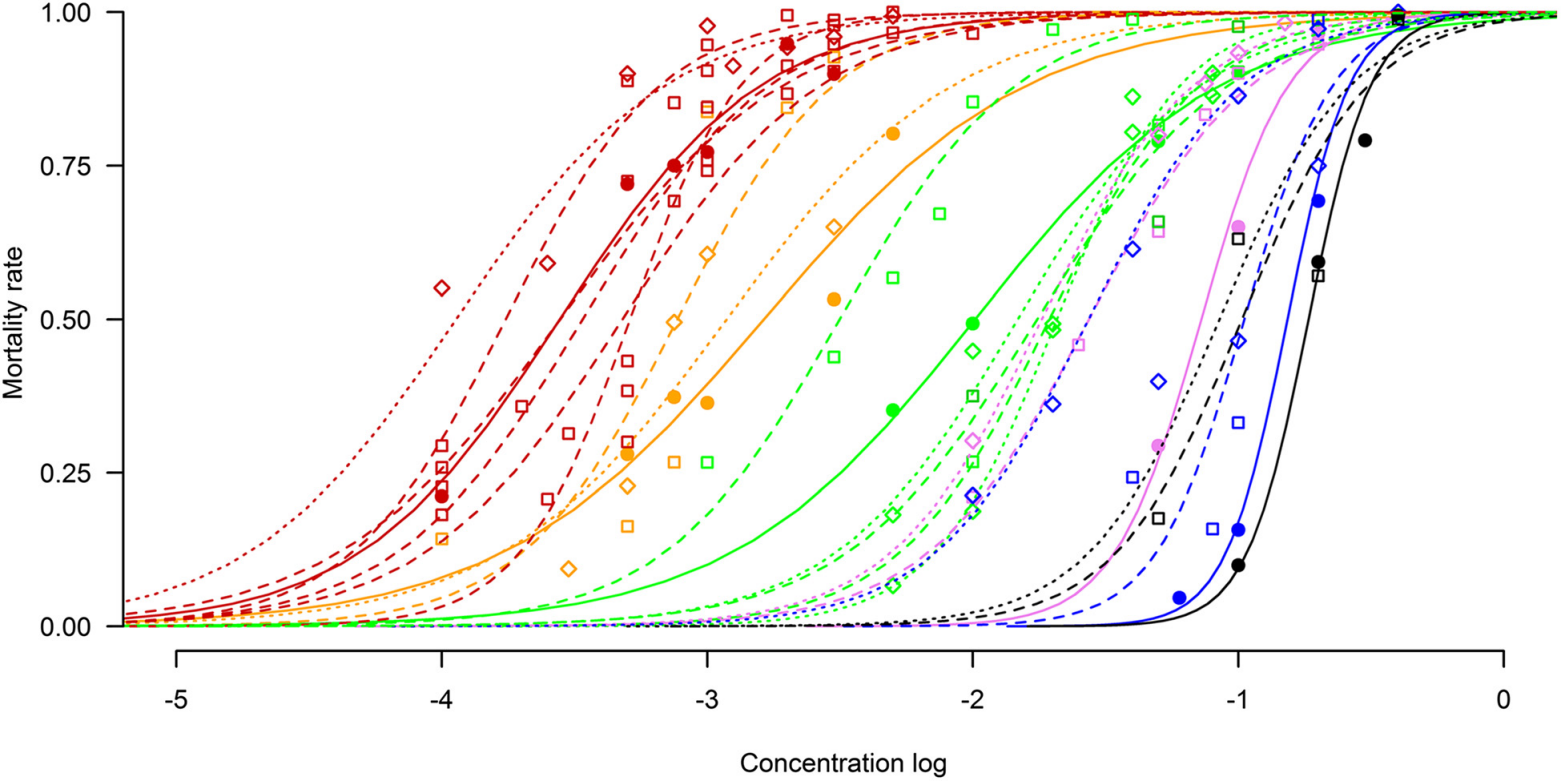
Sigmoidal curves of concentration-response estimations of different *Culicoides* populations exposed to different insecticides active ingredients. Dots represent pooled data obtained for each tested concentration (filled circle = *C. nubeculosus*; square = *C. obsoletus*; diamond = *C. imicola*) and lines represent the logistic regression of each population (straight: *C. nubeculosus,* dotted: *C. obsoletus*; dashed: *C. imicola*) for each active ingredient (red = deltamethrin; orange = alpha-cypermethrin; green = permethrin; purple = chlorpyriphos-methyl; blue = phoxim; black = diazinon). Data was analysed with PriProbit ver. 1.63, based on the mortality recorded at 24 h after 1 h exposure to different concentrations of active ingredients

Sigmoidal curves of dose–response obtained after data analysis indicated that SPs were 1–3 log-fold more effective/unit weight than OPs (Fig. 1). Within SPs, there was no significant impact according to the species tested for deltamethrin (*p* = 0.14), alpha-cypermethrin (*p* = 0.26) or permethrin (*p* = 0.65). In contrast, within OPs tested significant species effects were recorded in all three cases (*p* < 0.001). Chlorpyriphos-methyl and diazinon elicited LC_50_ values in *C. nubeculosus* which were 61.5–73.7 and 44.5–54.6 % higher than the other two species tested, whereas the phoxim LC_50_ of *C. obsoletus* was 74.4–82.1 % lower than the other two species (Fig. 1). Diagnostic concentrations (defined as twice the value of LC_99_) for the insecticides tested are presented in Table 5 and Additional file 6: S6. In addition, the statistical analysis showed that there was no difference between species in the diagnostic concentrations for deltamethrin (*p* = 0.98), alpha-cypermethrin (*p* = 0.26) or permethrin (*p* = 0.16). In contrast, OP active ingredients were significantly different in species effects (*p* < 0.001) between chlorpyriphos-methyl, phoxim and diazinon.

**Table 5:** Insecticide diagnostic concentrations expressed in % of active ingredient of different populations of *Culicoides* to different active ingredients

Active ingredient	Population (origin)						
	*C. nubeculosus*	*C. obsoletus*		*C. imicola*			
	(Cirad, FR)	(Corrèze, FR)	(Mallorca, ES)	(Corsica, FR)	(Catalonia, ES)	(Rufisque, SEN)	(Pretoria,SA)
Pyrethroids							
Deltamethrin	0.03	0.01	0.04	0.01	0.04	0.01	0.03
Alpha-cypermethrin	0.61	0.21		0.03			
Permethrin	2.65	0.48	1.12	0.77	1.55	0.20	
Organophosphates							
Chlorpyrifos-methyl	0.82	0.74		1.36			
Phoxim	1.05	1.16		1.22			
Diazinon	1.26	2.29		2.54			

Most of diagnostic concentrations likely overestimated due to classical dose/response analysis (see discussion)

*FR* France, *ES* Spain, *SEN* Senegal, *SA* South Africa

**Fig. 2:**
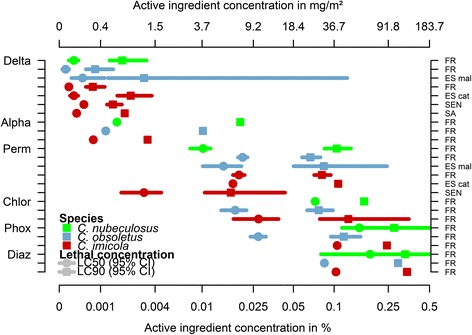
Lethal concentrations LC_50_, LC_90_ and 95 % confidence intervals for different populations exposed to six active ingredients: deltamethrin (Delta); alpha-cypermethrin (Alpha), permethrin (Perm), Chlorpyriphos-methyl (Chlor); phoxim (Phox) and diazinon (Diaz). Populations origin FR: France; ES mal: Mallorca Island, Spain; ES cat: Catalonia, Spain; SA: South Africa and SEN: Senegal. Lethal concentrations were calculated with PriProbit ver. 1.63, based on the mortality recorded at 24 h after 1 h exposure to different concentrations

When the effect of the variable origin (country) was tested, a significant effect was found for deltamethrin (p <0.001) and permethrin (p <0.001). LC_50_ and LC_90_ values of field-collected *Culicoides* populations from France to deltamethrin were significantly lower than those from other countries (Tables 1, 2, 3, 4). Similarly, when the effect of origin population was tested, a significant effect was found for deltamethrin (*p* < 0.001) and permethrin (*p* < 0.001) responses with the French population of *C. obsoletus* and the Senegal population of *C. imicola* eliciting lower LC_50_ and LC_90_ values for deltamethrin and permethrin, respectively (Figs. 1 and 2).

A steeper slope of sigmoidal curves (Fig. 1), and the small gap between LC_50_ and LC_90_ for deltamethrin and permethrin (Fig. 2), demonstrated the efficacy of pyrethroids to induce higher mortality in *Culicoides* by only a slight increase in dose. Despite the general robustness and low variability of the response within each population against each active ingredient, which can be inferred from the small size of gaps between lower and upper 95 % confidence intervals (95 % CI) (length of LC lines in Fig. 2), the results from the *C. obsoletus* population in Mallorca (Spain) were highly variable.

## Discussion

This study presents the first systematic survey of insecticide susceptibility of C*ulicoides* species of veterinary interest on a wide geographic scale in Europe and Africa. More than 25,000 *Culicoides* were tested to obtain a robust and reliable dataset demonstrating that species within the genus varied significantly in their susceptibility to SP and OP active ingredients according to their origin. The use of the standardized method enabled the assessment of the *Culicoides* susceptibility to current insecticides, providing important baseline information, including reference values for the laboratory reared-species, *C. nubeculosus*. SPs were more toxic to the three *Culicoides* species tested than OPs, confirming previous studies conducted in wind tunnel trials carried out in USA with other *Culicoides* vector species [27–29]. This differential toxicity between insecticide families has also previously been highlighted in other insect species such as mosquitoes [30–32] and termites [33].

The most toxic insecticides tested against *Culicoides* in the study were deltamethrin and alpha-cypermethrin, both of which are synthetic 2nd generation type II α-cyano SPs. During our trials, no excito-repellency effect was observed, even when *Culicoides* were exposed to the highest concentration of permethrin, which has a documented repellence effect in mosquitoes [31]. Further work is required, however, to define methods of testing the impact of repellency versus toxicity in *Culicoides* as this factor might influence their efficacy in the field.

The WHO recommendations for adult mosquito susceptibility tests advises the use of insects of standardized ages for analysis [19, 22]. This restricts material in use to either adult females derived from larval collections (the preferred option) or, if larval collections are not possible, the F_1_ progeny of field collected females. These cohorts were not used in the present study as colonization of *C. imicola* or *C. obsoletus* has not been achieved and larval rearing does not provide sufficient numbers of adults for use [34]. As a more logistically feasible alternative to circumvent this issue, field-collected unpigmented females were assumed to be of a similar age [23]. While this raises the issues of repeatability of the study when a population with a different age structure is assessed, the restriction to unpigmented individuals at least reduces this source of error to a likely variation of days rather than weeks, which could occur if both unpigmented and pigmented individuals were used. These issues highlight the requirement for further development of accurate age grading methods for *Culicoides,* which, at present, are lacking.

The classical approach (probit regression analysis) for assessment of dose–response data as LC_50_, LC_90_ and their 95 % confidence intervals has been used for decades [19]. However, the probit regression method used in the current study for data analysis of mortality data has limitations. Mainly, this method doesn’t take into account over-dispersion of the data and when this occurs it cannot calculate associated confidence intervals. The probit method also predicts beyond data observed limits and outputs reference values as LC_90_ or LC_99_ using this prediction, which may underestimate the effect of insecticides. Two examples illustrate this point: i) using 0.005 % of deltamethrin, 99 % of exposed *C. obsoletus* died, but the prediction for the LC_99_ was 0.007 % (1.4-fold greater than the observed data); ii) a 99 % mortality was observed with 0.4 % of permethrin, but the prediction was LC_99_ = 1.32 % (3.3-fold greater than the observed data). Thirdly, results obtained after analyses are aggregated and comparison between individual trials is difficult. This is in part due to the fact that the probit regression method was designed to demonstrate if a field population of a given species is less sensitive than a susceptible reference population of the same species when both populations are exposed to the same range of concentrations of an active ingredient.

The advantage of the second method of analysis used was that over-dispersion in the datasets is taken into account in the calculation method. In addition, the predictions remain inside the data limits, no pre-determined shape was imposed on the regressions and results are more detailed. This approach highlighted no species-specific differences in toxicity of the three SP active ingredients tested. Results with OPs highlighted species effects, however, suggesting natural species-specific susceptibility as previously reported for mosquitoes [35, 36]. This could also represent specific resistance mechanisms, although diagnostic concentrations are similar to those of susceptible mosquito strains of *Anopheles gambiae* [22].

Resistance to insecticides has been reported in the New World for *Haematobia irritans irritans* Linnaeus (Diptera: Muscidae), a large biting fly which has been specifically targeted by insecticide treatment of ruminants using pour-on applications. This resistance has been demonstrated to occur through several complex resistance mechanisms, including target site insensitivity [37, 38] and metabolic detoxification [39]. In order to detect the development of resistance mechanisms in *Culicoides*, as in mosquitoes, it is recommended to test the vector susceptibility by bioassay over time throughout the year to assess temporal trends in resistance, and also to compare multiple sites in order to assess geographical distribution of resistance. Similar surveys of broadly distributed species such as *C. imicola* and *C. obsoletus* could provide helpful information about resistance according to the different insecticide pressure and environmental context across countries. While *Culicoides* populations investigated in the current study present no evidence of resistance, low variability between results was observed except for the Mallorca (Spain) population of *C. obsoletus*. A key consideration is that multicentric insecticide trials are subject to extrinsic factors that can influence results, misleading the real effect of an insecticide to a given population [22, 40]. In the current study significant efforts were made to control conditions during testing including ambient temperature and humidity, the origin of impregnated papers and the bioassay procedure. However, one could not exclude the possibility that some differences in the procedures persist between countries.

## Conclusions

In conclusion, this study has defined the baseline susceptibility status of different *Culicoides* vector populations against six SP and OP insecticide active ingredients. The information regarding lethal concentrations will be used in future studies with the aim of testing the efficiency of insecticidal products applied directly on animals (e.g. pour-on and baths/dips), or insecticide impregnated materials (e.g. nets and paints) and also to monitor the potential emergence of resistance in field populations. This will improve our understanding of the efficacy of control measures against *Culicoides* in the field and enable better policy recommendations for their use in Europe and Africa.

## Additional files

Additional file 1: Table S1.
*Culicoides* collections sites and date of collection during trials. (DOCX 16 kb) Additional file 2: Table S2. Serial dilutions of insecticides used in trials to assess susceptibility of different populations of *Culicoides*. (DOCX 16 kb) Additional file 3: Table S3. Susceptibility values (LC_50_ and LC_90_ expressed in mg/m² of active ingredient) of *Culicoides nubeculosus* from French colony to different active ingredients. Mortality recorded 24 h after 1 h exposure to different concentrations. (DOCX 15 kb) Additional file 4: Table S4. Susceptibility values (LC_50_ and LC_90_ expressed in mg of active ingredient/m²) of different populations of *Culicoides obsoletus* to different active ingredients. Mortality was recorded 24 h after a 1 h exposure to insecticides. (DOCX 16 kb) Additional file 5: Table S5a. Susceptibility values (LC_50_ and LC_90_ expressed in mg of active ingredient/m²) of different European populations of *Culicoides imicola* to different active ingredients. Mortality was recorded 24 h after 1 h exposure to different concentrations. Table S5b. Susceptibility values (LC_50_ and LC_90_ expressed in mg of active ingredient/m²) of different African populations of *Culicoides imicola* to different active ingredients. Mortality was recorded 24 h after 1 h exposure to different concentrations. (DOC 54 kb) Additional file 6: Table S6. Insecticide diagnostic concentrations expressed in mg of active ingredient/m² of different populations of *Culicoides* to different active ingredients*. (DOCX 16 kb)*
