# Supplementary material for: How do species, population and active ingredient influence insecticide susceptibility in Culicoides biting midges (Diptera: Ceratopogonidae) of veterinary importance?
Source: Parasit Vectors. 2015 Aug 28;8:439. doi: 10.1186/s13071-015-1042-8 (PMC4551713; doi:10.1186/s13071-015-1042-8)
Supplement: Additional file 1: Table S1. — Culicoides collections sites and date of collection during trials. (DOCX 16 kb) [file 13071_2015_1042_MOESM1_ESM.docx]

**Table S1. *Culicoides* collections sites and date of collection during trials.**

| **Species** | **Origin (Country)** | **Location** | **Date of collection** |
| --- | --- | --- | --- |
| *Culicoides nubeculosus* | France | Cirad colony, Montpellier |  |
| *Culicoides imicola* | France | Piannotoli Caldarello, Corsica Island | 08/2012; 09/2013 |
|  | Spain | Caldes de Malavella, Catalonia | 09/2012 |
|  | Senegal | Niague, Rufisque | 03/2013, 02/2014 |
|  | South Africa | ARC-OVI Institute, Pretoria | 06/2010 |
| *Culicoides obsoletus* | France | Peret–Bel-Air, Corrèze | 06/2012, 06/2013 |
|  | Spain | Susqueda, Catalonia | 09/2010 |
|  | Spain | Felanitx, Mallorca Island | 06/2013 |
