# Supplementary material for: How do species, population and active ingredient influence insecticide susceptibility in Culicoides biting midges (Diptera: Ceratopogonidae) of veterinary importance?
Source: Parasit Vectors. 2015 Aug 28;8:439. doi: 10.1186/s13071-015-1042-8 (PMC4551713; doi:10.1186/s13071-015-1042-8)
Supplement: Additional file 2: Table S2. — Serial dilutions of insecticides used in trials to assess susceptibility of different populations of Culicoides. (DOCX 16 kb) [file 13071_2015_1042_MOESM2_ESM.docx]

**Table S2. Serial dilutions of insecticides used in trials to assess susceptibility of different populations of *Culicoides.***

| **Species** | **Origin (Country)** | **Active ingredient** | **Concentrations (%)** |
| --- | --- | --- | --- |
| *C. nubeculosus* | CIRAD colony (France) | Alpha-cypermethrin | 0.0001, 0.0005, 0.00075, 0.001, 0.003, 0.005 |
|  |  | Deltamethrin | 0.0001, 0.0005, 0.00075, 0.001, 0.002, 0.003 |
|  |  | Permethrin | 0.05, 0.01, 0.05, 0.1, 0.2, 0.4 |
|  |  | Chlorpyriphos-methyl | 0.05, 0.075, 0.1, 0.2 |
|  |  | Phoxim | 0.06, 0.1, 0.2, 0.4 |
|  |  | Diazinon | 0.1, 0.2, 0.3, 0.4, 0.5 |
| *C. imicola* | Corsica Island (France) | Alpha-cypermethrin | 0.0001, 0.0005, 0.00075, 0.001, 0.002, 0.003 |
|  |  | Deltamethrin | 0.0001, 0.0005, 0.00075, 0.001, 0.002, 0.003 |
|  |  | Permethrin | 0.01, 0.05, 0.1, 0.2, 0.4 |
|  |  | Chlorpyriphos-methyl | 0.01, 0.025, 0.05, 0.075, 0.1, 0.2 |
|  |  | Phoxim | 0.04, 0.08, 0.1, 0.2 |
|  |  | Diazinon | 0.005, 0.001, 0.05, 0.1, 0.2 |
|  | Catalonia (Spain) | Deltamethrin | 0.0001, 0.0005, 0.001, 0.002, 0.003, 0.005 |
|  |  | Permethrin | 0.001, 0.005, 0.01, 0.05, 0.1 |
|  | Rufisque (Senegal) | Deltamethrin | 0.00025, 0.0005, 0.00075, 0.001, 0.002, 0.003 |
|  |  | Permethrin | 0.001, 0.003, 0.005, 0.0075, 0.01, 0.02, 0.04 |
|  | Pretoria (RSA) | Deltamethrin | 0.0001, 0.0005, 0.001, 0.005, 0.01 |
| *C. obsoletus* | Corrèze (France) | Alpha-cypermethrin | 0.0003, 0.0005, 0.00075, 0.001, 0.003 |
|  |  | Deltamethrin | 0.0001, 0.00025, 0.0005, 0.001, 0.00125, 0.002, 0.003, 0.005 |
|  |  | Permethrin | 0.01, 0.02, 0.04, 0.08 |
|  |  | Chlorpyriphos-methyl | 0.01, 0.05, 0.075, 0.1, 0.15 |
|  |  | Phoxim | 0.01, 0.02, 0.04, 0.1, 0.2 |
|  |  | Diazinon | 0.05, 0.1, 0.2, 0.4 |
|  | Mallorca (Spain) | Deltamethrin | 0.0001, 0.0002, 0.0003, 0.0005, 0.001, 0.003 |
|  |  | Permethrin | 0.005, 0.01, 0.02, 0.04, 0.08 |
