# Supplementary material for: How do species, population and active ingredient influence insecticide susceptibility in Culicoides biting midges (Diptera: Ceratopogonidae) of veterinary importance?
Source: Parasit Vectors. 2015 Aug 28;8:439. doi: 10.1186/s13071-015-1042-8 (PMC4551713; doi:10.1186/s13071-015-1042-8)
Supplement: Additional file 3: Table S3. — Susceptibility values (LC50 and LC90 expressed in mg/m² of active ingredient) of Culicoides nubeculosus from French colony to different active ingredients. Mortality recorded 24 h after 1 h exposure to different concentrations. (DOCX 15 kb) [file 13071_2015_1042_MOESM3_ESM.docx]

**Table S3.** **Susceptibility values (LC_50_ and LC_90_ expressed in mg/m² of active ingredient) of *Culicoides* *nubeculosus* from French colony to different active ingredients. Mortality recorded 24h after 1h exposure to different concentrations.**

| **Active ingredient** | **No. test** | **LC_50_ (mg/m²)** | **LC_90_ (mg/m²)** |
| --- | --- | --- | --- |
|  | **(n)** | **(95% CI)** | **(95% CI)** |
| Deltamethrin | 4 | 0.10 | 0.69 |
|  | (2,528) | (0.05-0.15) | (0.45-1.59) |
| Alpha-cypermethrin | 3 | 0.59 | 7.31 |
|  | (1,883) | NA | NA |
| Permethrin | 3 | 3.75 | 38.39 |
|  | (2,055) | (3.23-4.28) | (31.19-49.11) |
| Chlorpyrifos-methyl | 4 | 26.64 | 61.02 |
|  | (1,803) | NA | NA |
| Phoxim | 3 | 56.26 | 101.35 |
|  | (1,965) | (41.98-76.81) | (74.78-211.62) |
| Diazinon | 4 | 67.55 | 121.85 |
|  | (1,527) | (29.38-100.91) | (86.22-159.17) |

No. test = number of test performed. *n* = number of individual tested. CI = confidence interval, NA = confidence interval not computed due to a large variability in the dose/response effect.
