# Supplementary material for: How do species, population and active ingredient influence insecticide susceptibility in Culicoides biting midges (Diptera: Ceratopogonidae) of veterinary importance?
Source: Parasit Vectors. 2015 Aug 28;8:439. doi: 10.1186/s13071-015-1042-8 (PMC4551713; doi:10.1186/s13071-015-1042-8)
Supplement: Additional file 4: Table S4. — Susceptibility values (LC50 and LC90 expressed in mg of active ingredient/m²) of different populations of Culicoides obsoletus to different active ingredients. Mortality was recorded 24 h after a 1 h exposure to insecticides. (DOCX 16 kb) [file 13071_2015_1042_MOESM4_ESM.docx]

**Table S4.** **Susceptibility values (LC_50_ and LC_90_ expressed in mg of active ingredient/m²) of different populations of *Culicoides obsoletus* to different active ingredients. Mortality was recorded 24h after a 1h exposure to insecticides.**

|  | ***C. obsoletus* (Corrèze, France)** | | | ***C. obsoletus* (Mallorca, Spain)** | | | |
| --- | --- | --- | --- | --- | --- | --- | --- |
| **Active ingredient** | **No. test** | **LC_50_ (mg/m²)** | **LC_90_ (mg/m²)** | | **No. test** | **LC_50_ (mg/m²)** | **LC_90 (_mg/m²)** |
|  | **(n)** | **(95% CI)** | **(95% CI)** | | **(n)** | **(95% CI)** | **(95% CI)** |
| Deltamethrin | 3 | 0.04 | 0.30 | | 4 | 0.18 | 1.18 |
|  | (1,491) | (0.01-0.07) | (0.19-0.37) | | (382) | (0.07-0.40) | (0.48-45.77) |
| Alpha-cypermethrin | 3 | 0.43 | 3.73 | |  |  |  |
|  | (502) | NA | NA | |  |  |  |
| Permethrin | 2 | 7.62 | 24.53 | | 4 | 5.40 | 30.86 |
|  | (527) | (6.91-8.42) | (20.75-30.21) | | (131) | (3.71-7.46) | (18.44-89.81) |
| Chlorpyrifos-methyl | 5 | 6.67 | 28.23 | |  |  |  |
|  | (615) | (5.20-8.15) | (23.20-35.98) | |  |  |  |
| Phoxim | 6 | 10.01 | 37.58 | |  |  |  |
|  | (763) | (8.74-11.45) | (29.57-47.75) | |  |  |  |
| Diazinon | 2 | 31.16 | 108.13 | |  |  |  |
|  | (917) | NA | NA | |  |  |  |

No. test = number of test performed. *n* = number of individual tested. CI = confidence interval, NA = confidence interval not computed, due to a large variability in the dose/response effect.
