# Supplementary material for: How do species, population and active ingredient influence insecticide susceptibility in Culicoides biting midges (Diptera: Ceratopogonidae) of veterinary importance?
Source: Parasit Vectors. 2015 Aug 28;8:439. doi: 10.1186/s13071-015-1042-8 (PMC4551713; doi:10.1186/s13071-015-1042-8)
Supplement: Additional file 5: Table S5a. — Susceptibility values (LC50 and LC90 expressed in mg of active ingredient/m²) of different European populations of Culicoides imicola to different active ingredients. Mortality was recorded 24 h after 1 h exposure to different concentrations. Table S5b. Susceptibility values (LC50 and LC90 expressed in mg of active ingredient/m²) of different African populations of Culicoides imicola to different active ingredients. Mortality was recorded 24 h after 1 h exposure to different concentrations. (DOC 54 kb) [file 13071_2015_1042_MOESM5_ESM.doc]

**Table S5a.** **Susceptibility values (LC_50_ and LC_90_ expressed in mg of active ingredient/m²) of different European populations of *Culicoides imicola* to different active ingredients. Mortality was recorded 24h after 1h exposure to different concentrations.**

|  | **Corsica, France** | | | **Catalonia, Spain** | | |
| --- | --- | --- | --- | --- | --- | --- |
| **Active ingredient** | **No. test** | **LC_50_ (mg/m²)** | **LC_90_ (mg/m²)** | **No. test** | **LC_50_ (mg/m²)** | **LC_90_ (mg/m²)** |
|  | **(n)** | **(95%CI)** | **(95%CI)** | **(n)** | **(95%CI)** | **(95%CI)** |
| Deltamethrin | 3 | 0.06 | 0.28 | 3 | 0.11 | 0.84 |
|  | (2,525) | (0.04-0.09) | (0.19-0.49) | (378) | (0.07-0.15) | (0.59-1.36) |
| Alpha-cypermethrin | 4 | 0.28 | 1.25 |  |  |  |
|  | (2.084) | NA | NA |  |  |  |
| Permethrin | 4 | 7.14 | 29.84 | 2 | 6.43 | 39.34 |
|  | (1.562) | (6.29-8.04) | (25.54-35.81) | (123) | NA | NA |
| Chlorpyrifos-methyl | 1 | 10.06 | 46.75 |  |  |  |
|  | (122) | (6.47-14.24) | (28.81-129.84) |  |  |  |
| Phoxim | 2 | 38.68 | 89.68 |  |  |  |
|  | (2,207) | NA | NA |  |  |  |
| Diazinon | 2 | 37.89 | 125.78 |  |  |  |
|  | (1,704) | NA | NA |  |  |  |

No. test = number of test performed. *n* = number of individual tested. CI = confidence interval, NA = confidence interval not computed, due to a large variability in the dose/response effect.

**Table S5b.** **Susceptibility values (LC_50_ and LC_90_ expressed in mg of active ingredient/m²) of different African populations of *Culicoides imicola* to different active ingredients. Mortality was recorded 24h after 1h exposure to different concentrations.**

|  | **Rufisque, Senegal** | | | **Pretoria, South Africa** | | |
| --- | --- | --- | --- | --- | --- | --- |
| **Active ingredient** | **No. test** | **LC_50_ (mg/m²)** | **LC_90_ (mg/m²)** | **No. test** | **LC_50 (_mg/m²)** | **LC_90(_ mg/m²)** |
|  | **(n)** | **(95%CI)** | **(95%CI)** | **(n)** | **(95%CI)** | **(95%CI)** |
| Deltamethrin | 4 | 0.18 | 0.55 | 3 | 0.11 | 0.73 |
|  | (458) | (0.15-0.18) | (0.44-0.66) | (291) | NA | NA |
| Permethrin | 3 | 1.14 | 6.17 |  |  |  |
|  | (521) | (0.66-1.65) | (3.86-15.83) |  |  |  |

No. test = number of test performed. *n* = number of individual tested. CI = confidence interval, NA = confidence interval not computed, due to a large variability in the dose/response effect.
