# Supplementary material for: How do species, population and active ingredient influence insecticide susceptibility in Culicoides biting midges (Diptera: Ceratopogonidae) of veterinary importance?
Source: Parasit Vectors. 2015 Aug 28;8:439. doi: 10.1186/s13071-015-1042-8 (PMC4551713; doi:10.1186/s13071-015-1042-8)
Supplement: Additional file 6: Table S6. — Insecticide diagnostic concentrations expressed in mg of active ingredient/m² of different populations of Culicoides to different active ingredients. (DOCX 16 kb) [file 13071_2015_1042_MOESM6_ESM.docx]

**Table S6.** **Insecticide diagnostic concentrations expressed in mg of active ingredient/m² of different populations of *Culicoides* to different active ingredients*.***

| **Active ingredient** | **Population (origin)** | |  | | | | |
| --- | --- | --- | --- | --- | --- | --- | --- |
|  | ***C. nubeculosus*** | ***C. obsoletus*** |  | ***C. imicola*** |  |  |  |
|  | **(Cirad, FR)** | **(Corrèze, FR)** | **(Mallorca, ES)** | **(Corsica, FR)** | **(Catalonia, ES)** | **(Rufisque, SEN)** | **Pretoria (SA)** |
| **Pyrethroids** |  |  |  |  |  |  |  |
| Deltamethrin | 11.02 | 3.67 | 14.69 | 3.67 | 14.69 | 3.67 | 11.02 |
| Alpha-cypermethrin | 224.08 | 77.14 |  | 11.02 |  |  |  |
| Permethrin | 973.45 | 176.32 | 411.42 | 282.85 | 569.38 | 73.47 |  |
| **Organophosphates** |  |  |  |  |  |  |  |
| Chlorpyrifos-methyl | 301.22 | 271.83 |  | 499.58 |  |  |  |
| Phoxim | 385.71 | 426.11 |  | 448.15 |  |  |  |
| Diazinon | 462.85 | 841.21 |  | 933.04 |  |  |  |

FR = France, ES = Spain, SEN = Senegal, SA = South Africa.

Notice: most of diagnostic concentrations likely overestimated due to classical dose/response analysis (see discussion).
